# A novel beneficial role of humanin on intestinal apoptosis and dysmotility in a rat model of ischemia reperfusion injury

**DOI:** 10.1007/s00424-023-02804-0

**Published:** 2023-04-05

**Authors:** Eman R. Abozaid, Reham H. Abdel-Kareem, Marwa A. Habib

**Affiliations:** 1 Medical Physiology Department, Faculty of Medicine, Zagazig University, Alsharquiah, 44519 Egypt; 2 Human Anatomy & Embryology Department, Faculty of Medicine, Zagazig University, Alsharquiah, 44519 Egypt

**Keywords:** Humanin, Intestinal motility, Ischemia/reperfusion, Nitric oxide

## Abstract

A prevalent clinical problem including sepsis, shock, necrotizing enterocolitis, and mesenteric thrombosis is intestinal ischemia/reperfusion (I/R) injury. Humanin (HN), a recently identified mitochondrial polypeptide, exhibits antioxidative and antiapoptotic properties. This work aimed to study the role of HN in a model of experimental intestinal I/R injury and its effect on associated dysmotility. A total of 36 male adult albino rats were allocated into 3 equal groups. Sham group: merely a laparotomy was done. I/R group: for 1 h, clamping of the superior mesenteric artery was done, and then reperfusion was allowed for 2 h later. HN-I/R group: rats underwent ischemia and reperfusion, and 30 min before the reperfusion, they received an intraperitoneal injection of 252 μg/kg of HN. Small intestinal motility was evaluated, and jejunal samples were got for biochemical and histological analysis. I/R group showed elevation of intestinal NO, MDA, TNF- α, and IL-6 and decline of GPx and SOD levels. Furthermore, histologically, there were destructed jejunal villi especially their tips and increased tissue expression of caspase-3 and i-NOS, in addition to reduced small intestinal motility. Compared to I/R group, HN-I/R group exhibited decrease intestinal levels of NO, MDA, TNF- α, and IL-6 and increase GPx and SOD. Moreover, there was noticeable improvement of the histopathologic features and decreased caspase-3 and iNOS immunoreactivity, beside enhanced small intestinal motility. HN alleviates inflammation, apoptosis, and intestinal dysmotility encouraged by I/R. Additionally, I/R-induced apoptosis and motility alterations depend partly on the production of nitric oxide.

## Introduction

Intestinal ischemia/reperfusion (I/R) injury is involved in numerous clinical issues, including shock, sepsis, necrotizing enterocolitis, and mesenteric thrombosis [2, 12, 46]. Although ischemia–reperfusion injury can affect any organ, the I/R of the intestine has the highest morbidity and mortality rates [6]. This is due to its propensity to impact almost all organs, which can result in multiple-organ failure [16, 23]. Likewise, an abrupt interruption of blood flow to the intestine, followed by blood flow restoration, causes oxidative stress, inflammation, and cell apoptosis [47].

Intestinal I/R affects the intestine’s motility [47, 56, 62]. Also, I/R-related motility changes impair intestinal mucosa and barrier function, impair immunity, allow intestinal bacteria and endotoxins to spread throughout the body, and even result in multiple organ dysfunction syndrome [67, 71].

It has been demonstrated that intestinal I/R causes an increase in nitric oxide synthase (NOS) [30, 44]. Nitric oxide (NO) role in intestinal I/R is unclear and complicated. It is controversial whether NO has cytotoxic or cytoprotective properties because of the variety of functions it plays in the body. It is understood that NO keeps a balance between vasoconstriction and vasodilatation [37]. The modulation of smooth muscle contractions by NO via nonadrenergic noncholinergic neurons is essential [45]. Therefore, changes in the basal level of NO or I/R-induced NO generation may affect intestinal motility.

A 24-amino acid mitochondrial polypeptide called humanin (HN) has anti-apoptotic [35] and neuroprotective properties [73]. Numerous studies have demonstrated that it could be able to stop cell damage caused by stress or injury in a variety of tissues including the neurological system, cardiovascular system, pancreas, and bone [20, 53, 55]. By regulating the intrinsic mitochondrial pathway and the expression of intracellular apoptotic and antiapoptotic markers, HN displays antiapoptotic action in addition to its cytoprotective effects [53].

HN restored viability and cell proliferation by decreasing oxidative stress and NO, and it also increased the level of antioxidants in human neuroblastoma cells [33]. Moreover, HN-reduced N-Methyl-D-aspartate (NMDA) encouraged oxidative stress and NO generation in cultured rat cortical neurons [19]. Collectively, these findings direct that HN may be able to trigger a protective reaction against oxidative damage.

Although it has been claimed that HN guards against I/R damage to the heart and brain tissues, however, its effect on intestinal I/R injury and its associated dysmotility has not been clarified yet. So this present study was directed to examine the role of HN in a model of experimental intestinal I/R injury and its effect on associated dysmotility.

## Material and methods

### Animal experiment

Between October and November 2022, 36 healthy male adult albino rats of the local strain (210–250 g) were utilized in the study at Zagazig scientific and medical research center (ZSMRC), Faculty of Medicine, Zagazig University. The rats were got from the animal house of the Zagazig Veterinary Medicine Faculty. Rats were housed in five-rat steel wire cages at room temperature with a natural light/dark cycle, free access to water, and hygienic conditions for 1 week preceding the beginning of the study. The Institutional Animal Care and Use Committee of Zagazig University (IACUC) and the physiology department both gave their approval to the experimental protocol, with approval no (ZU-IACUC/3/F/219/2022).

### Experimental design

**Fig. 1:**
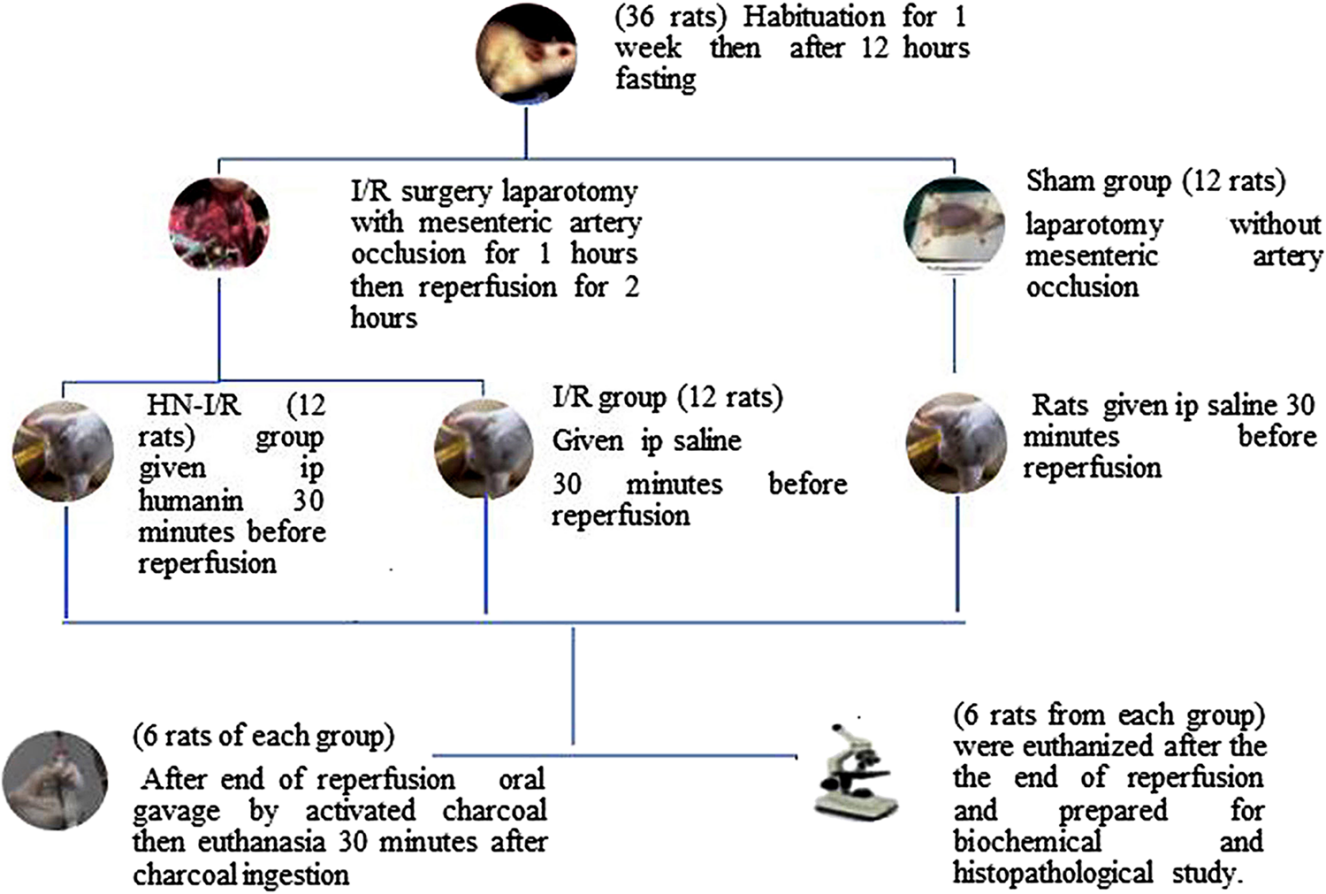
Experimental design flowchart

In accordance with the protocols used, as presented in our design flow chart (Fig. 1). After 1 week of acclimatization, rats were allocated randomly into three groups, each of which had twelve rats. After 12 h (h) fasting, 1st group (Sham group): merely a laparotomy was done, no superior mesenteric artery ligation, and no ischemia. To create the intestinal I/R injury model, surgery was done on the 2nd group (I/R group). For 1 h, clamping of the superior mesenteric artery was done, then reperfusion was allowed by removal of the clamp from the artery, and we maintained reperfusion for 2 h later. During the time of ischemia, 30 min prior to the reperfusion, rats of sham and I/R groups were injected intraperitoneally (i.p) by 0.1 mL of physiological saline as a vehicle. In the 3rd group (HN-I/R group), rats underwent superior mesenteric artery ligation (for 1 h) and reperfusion (for 2 h), but 30 min before the reperfusion, they received i.p injection of HN in a form of synthetic humanin analogue (HNG) peptide dissolved in saline in a dose of 252 μg/kg [64].

### Intestinal I/R injury model

The procedures were carried out using sterile techniques after a 12-h fast, as previously demonstrated by Fan et al. and Zan et al. [25, 72], with some modification. Rats were injected by 25 mg/kg thiopental sodium (i.p.), to make them unconscious before the operation. The abdomen was then sterilely prepared after the hair had been removed. To compensate for fluid loss, 1 mL of subcutaneous 0.9% saline was administered prior to surgery. Through a midline incision, a traumatic vascular clamp blocked the superior mesenteric artery. The abdomen was briefly sutured to stop fluid loss and the intestine was moved into the peritoneal cavity. Throughout the experiment, the rats were kept on heating pads at a temperature of 37 °C. After superior mesenteric artery closure, all rats received 25 ml/kg of 0.9% saline administered subcutaneously as resuscitation. Two hours after the reperfusion, rats in each group are equally divided, 6 rats were used for further intestinal motility assessment, and the other 6 rats were euthanized by 100 mg/kg thiopental sodium (i.p. injection) then jejunal samples were collected, half of the samples were immediately put at − 80 °C for tissue homogenate analysis and biochemical assay, and the other half was placed in 10% formalin solution for histopathologic and immunohistochemical examinations.

### Functional study of the small intestinal motility

A total of 0.5 ml volume of the charcoal meal (10% activated charcoal suspended in 5% aqueous gum arabic) was administered into each rat’s stomach through a gastric tube. The rats were then euthanized after 30 min by 100 mg/kg thiopental sodium (i.p. injection). The entire small intestine was extracted after laparotomy. The charcoal’s journey from the pylorus to its farthest point was measured. For calculation of the ratio of small intestine impelling force, the following formula was utilized: intestinal impelling force ratio (%) = distance covered by the charcoal/total small intestine length × 100 [39, 54].

### Intestinal tissue biomarkers assessment

The intestinal sample was centrifuged at 50,000 rpm for 15 min after being homogenized; cadmium reduction method modification was used to assess the supernatant. Using commercial test kits, these homogenates were utilized to evaluate the oxidative stress indices glutathione peroxidase (GPx), superoxide dismutase (SOD), and malondialdehyde (MDA) activities (MBS744364, MBS2707324, and MBS268427 MyBioSource, respectively). These analytical biochemical techniques use an HRP colorimetric detection system and antibody-antigen interactions (immunosorbency) to identify antigen targets in samples in accordance with the manufacturer’s instructions. Additionally, rat ELISA kits were utilized according to the manufacturer’s recommendations (Bio diagnostic, Egypt) to assess NO and inflammatory markers (TNF-α and IL-6) levels.

### Jejunal tissue preparation for histological and immunohistochemical examination

#### Histological examination

Fixation of jejunal tissue samples for 48 h in 10% neutral buffered formalin solution (pH: 7.4), dehydration in alcohol, insertion in paraffin wax, serially sectioned (5 μm thickness), and then stained with Hematoxylin and Eosin (H & E) [60].

#### Immunohistochemistry

##### Inducible nitric oxide synthase (iNOS) and caspase-3 immunostaining

Paraffin sections of 4–5 μm were deparaffinized in xylene and processed for caspase-3 and iNOS antibodies immunohistochemistry. Sections were rehydrated in a descending series of ethanol and washed in phosphate-buffered saline (PBS). Inactivation of endogenous peroxide via 10% hydrogen peroxide (Sigma) in PBS (pH 7.4) for 10 min. Sections were incubated with 1:100 dilution of anti-caspas3 (rabbit polyclonal anti-rat activated caspase-3 antibody (ab2302; Abcam, Cambridge, Massachusetts, USA) [8] and anti-iNOS (rabbit anti-rats iNOS polyclonal antibody, M-19/Sc 650, Santa Cruz Biotechnology, Santa Cruz, CA, USA) [4]. Incubation of sections for 30 min with biotinylated secondary antibody, after rinsing with PBS (3 times for 5 min/slide) was done, followed by streptavidin– peroxidase complex at room temperature for 10 min. After adding 0.05% diaminobenzidine (DAB) reagent, the antigen–antibody reaction could be seen. The material was dehydrated and mounted after counterstaining with Mayer’s Hematoxylin. The negative control sections were done by omitting the primary antibody. The positive control sections (brown color) were prepared for caspase-3 (cytoplasmic and nuclear reaction) and iNOS (cytoplasmic cellular reaction) expressions.

Slides were examined and photographed by an electric light microscope (Leica ICC50 W) at the Image Analysis Unit of the Human Anatomy and Embryology Department, Faculty of Medicine, Zagazig University.

#### Morphometric analysis

Image analysis software (ImageJ 1.36b; http://rsbweb.nih.gov/ij) was used for the estimation of jejunal villus length and width (μm) and area% of caspase-3 and iNOS expressions [31, 51]. Quantitative data was evaluated in non-overlapped random different fields in each slide (magnification 100 for villus length and width and magnification 400 for immunohistochemistry).

### Statistical analysis

The SPSS program (version 28) (SPSS Inc. Chicago, IL, USA) was utilized to statistically evaluate the data for this study. A one-way analysis of variance (ANOVA) was used followed by Tukey’s post-hoc test, with a *P*-value of 0.05 deemed statistically significant. The *P*-value of > 0.05 displayed non-significant results, the *P*-value of < 0.05 displayed significant results, and the *P*-value of < 0.001 displayed highly significant results.

## Results

### Effect of humanin and I/R injury on oxidative stress and inflammatory markers in the intestinal tissue

Small intestine MDA, GPx, and SOD concentrations are reliable markers of oxidative stress and ischemia injury. Compared with the sham group, I/R significantly reduced GPx and SOD levels while significantly increased intestinal tissue MDA (*p* < 0.001) (Table 1). Compared with the I/R group, in the HN-I/R group, humanin significantly reduced intestinal tissue MDA and had elevated GPx and SOD levels (*P* < 0.001). Compared with the sham, in the HN-I/R group, there were significant differences regarding MDA (*p* < 0.001) and GPx (*p* < 0.05), but no significant difference regarding SOD (*p* > 0.05) (Table 1). Moreover, in the I/R group, the intestinal levels of TNF-α, IL-6, and NO levels significantly rose (*p* < 0.001), as compared to the sham group. But the humanin treatment in the HN-I/R group markedly reduced these levels (*P* < 0.001) when compared to the I/R group (Table 1). However, compared with the sham group, in the HN-I/R group, there were significant differences regarding IL-6 (*p* < 0.001), TNF–α, and NO (*p* > 0.05).

**Table 1:** Intestinal MDA, SOD, GPX, TNF–α, and IL-6 and NO in all studied groups

Groups Parameters		Sham group	I/R group	HN–I/R group
MDA (nmol/g protein	$\overline{\rm X}$ ± SD	4.27 ± 0.43	9.33 ± 0.81^**^	6.63 ± 0.58 ^**, ##^
SOD (U/µg protein)	$\overline{\rm X}$ ± SD	4.22 ± 0.5	2.98 ± 0.43 ^**^	4.13 ± 0.4 ^##^
GPX (U/µg protein)	$\overline{\rm X}$ ± SD	131.02 ± 1.92	71.20 ± 2.33 ^**^	114.32 ± 8.65 ^*^^, ##^
TNFα (pg/mg tissue)	$\overline{\rm X}$ ± SD	25.00 ± 0.72	87.07 ± 0.88 ^**^	32.88 ± 4.72 ^*^^, ##^
IL-6 (pg/ mg tissue)	$\overline{\rm X}$ ± SD	37.02 ± 0.68	93.97 ± 0.98 ^**^	55.8 ± 3.11 ^**, ##^
NO (µmol/ mg tissue)	$\overline{\rm X}$ ± SD	50.93 ± 1.21	99.07 ± 1.25 ^**^	56.83 ± 4.04 ^*^^, ##^

^*^, significant versus sham group (*P* < 0.05); ^**^, highly significant versus sham group (*P* < 0.01); ^#^, significant versus I/R group (*P* < 0.05); and ^##^, highly significant versus I/R group (*P* < 0.001)

#### H&E histological results

**Fig. 2:**
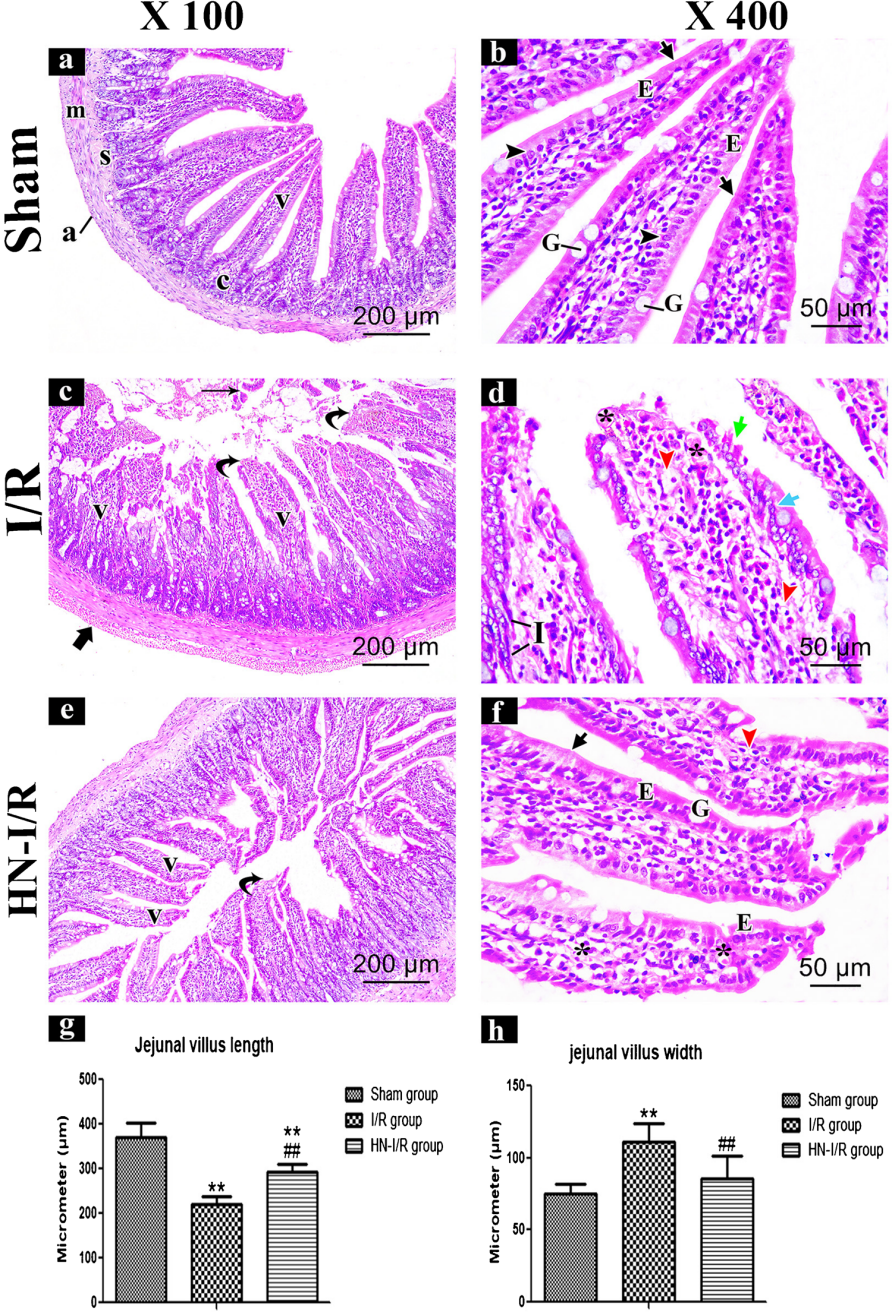
H&E stained sections of the jejunal wall of the sham group (**a** and **b**) is showing intact mucosa, submucosa (s), musculosa (m) and adventitia (a) layers. Jejunal mucosa has normally organized villi (v) and crypts (c). The villi are long slender with narrow tip, and covered by compactly packed tall columnar enterocytes (E) with noticeable goblet cells (G) in between. Enterocytes have continuous normal brush borders (short arrows) and basal vesicular nuclei (arrow heads). I/R group (**c** and **d**) is showing broad, shortened and disorganized jejunal villi (v) with desquamated tips (curved arrows), with presence of fragments cellular debris of detached villi in intestinal the lumen (thin arrow). Hemorrhage (*), mononuclear cellular infiltration (I), wide spaces and vacuolations (red arrow heads) are observed in lamina propria, moreover, enterocytes are disorganized (blue short arrow) and losing their brush borders (green short arrows). Also, there are abundant hemorrhage and thickening of adventitia layer (thick arrow). In HN-I/R group (**e** and **f**), few villi are still having desquamated tips (curved arrows), but most of villi (v) are restoring their length and structure with normal distribution of enterocytes (E), continuous brush borders (short arrow) and goblet cells (G). However, mild hemorrhage (*), vacuolations (red arrow head) of the lamina propria are still observed. (Bar: a&c&e = 200 μm X 100 and b,d&f = 50 μm X 400). **g** and **h** Morphometric comparing of jejunal villus length (**g**) and width (**h**) in all studied groups. Data are presented as mean ± SD, ^*^: *significant versus* sham group (*P* < 0.05), ^**^: highly significant versus sham group (*P* < 0.01), ^#^: significant versus I/R group (*P* < 0.05), and.^##^: highly significant versus I/R group (*P* < 0.001)

The sham group showed intact layers of the jejunal wall (mucosa, submucosa, musculosa, and adventitia). Jejunal mucosa showed normally organized villi and crypts. The villi were long, slender, with narrow tips, and covered by compactly packed tall columnar cells (enterocytes) with observable goblet cells in between. Enterocytes had continuous normal brush borders and basal vesicular nuclei (Fig. 2a–b).

The I/R group showed destructed villi, but crypt layers were still intact. Jejunal villi were broad, shortened, and disorganized with desquamated tips and had lost their covering epithelial cell, with the presence of fragments of cellular debris from detached villi in the intestinal lumen. Wide spaces, vacuolations, hemorrhage, and mononuclear cellular infiltration were observed in the villus core (lamina propria); moreover, the lining enterocytes were disorganized and lost their brush borders. Also, there were abundant hemorrhage and thickening of the adventitia layer (Fig. 2c–d).

The HN-I/R group showed noticeable improvement of histopathological signs of the inflammation and cellular infiltration compared with the I/R group. Few villi were still having desquamated tips, but most of the villi restored their length and structure with the normal distribution of enterocytes, continuous brush borders and goblet cells. However, mild hemorrhage and vacuolations of the lamina propria were still observed (Fig. 2e–f).

Morphometric analysis of jejunal villus length showed a significant reduction in the I/R group, as compared with the sham group (*p* < 0.001). However, in the HN-I/R group, villus length was significantly (decreased/increase) when comparing to (the sham group/I/R group) (*p* < 0.001), respectively (Fig. 2g).

Morphometric evaluation of the jejunal villus width revealed a significant increase in the I/R group, in comparison with the sham group (*p* < 0.001). While the villus width in the HN-I/R group showed no significant difference as compared with the sham group and a significant decrease as compared with the I/R group (*p* < 0.001) (Fig. 2h).

### Immunohistochemical results

#### Caspase-3 immunohistochemical results

**Fig. 3:**
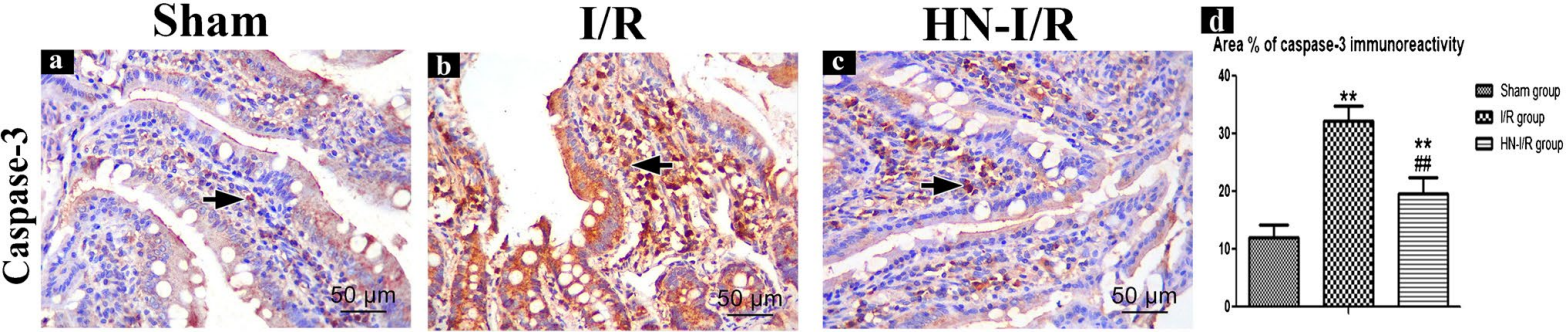
Immunoreactivity for caspase-3 in jejunal villi: positively stained cells are taking brown color (short arrow). **A** Sham group; **B** I/R group; **C** HN-I/R group; **D** morphometrical comparing of area percentage of caspase-3 immunoreactivity in all studied groups. Data are presented as mean ± SD, ^*^: significant versus sham group (*P* < 0.05), ^**^: highly significant versus sham group (*P* < 0.01), ^#^: significant versus I/R group (*P* < 0.05), and.^##^: highly significant versus I/R group (*P* < 0.001)

The villi core and the lining enterocytes of the sham group showed little positive cytoplasmic and nuclear immunoreactivity for caspase-3 (Fig. 3a), which was marked and diffuse in the I/R group (Fig. 3b) and moderately scattered in HN-I/R group (Fig. 3c).

Morphometric evaluation of the area percentage of caspase-3 immunoreactivity revealed a significant increase in the I/R group vs the sham group (*p* < 0.001). While, in the HN-I/R group, there was a significant (increase/decrease) of caspase-3 immunoreactivity vs (sham group/ I/R group), respectively (*p* < 0.001) (Fig. 3d).

#### iNOS immunohistochemical results

**Fig. 4:**
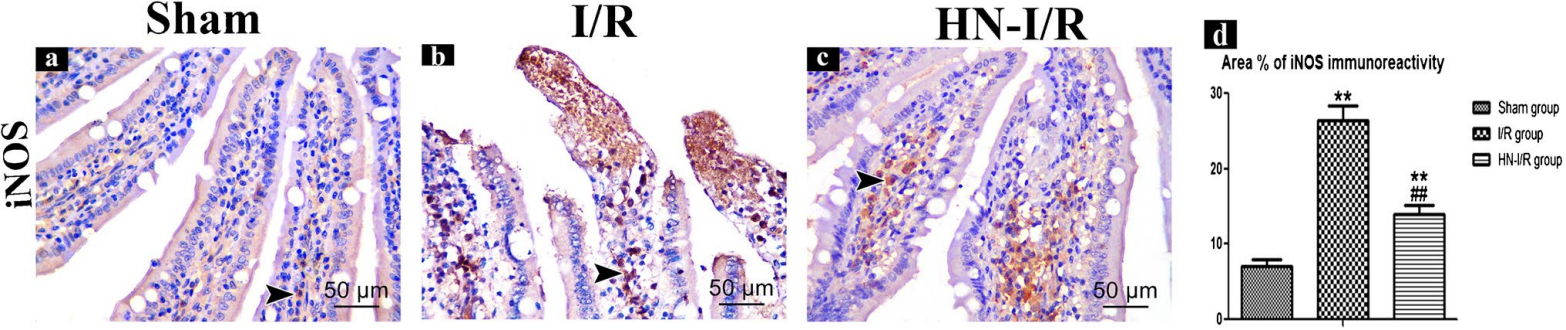
Immunoreactivity for iNOS in jejunal villi: positively stained cytoplasm is taking brown color (arrow head). **A** Sham group; **B** I/R group; **C** HN-I/R group; **D** morphometrical comparing of area percentage of iNOS immunoreactivity in all studied groups. Data are presented as mean ± SD, ^*^: significant versus sham group (*P* < 0.05), ^**^: highly significant versus sham group (*P* < 0.01), ^#^: significant versus I/R group (*P* < 0.05), and.^##^: highly significant versus I/R group (*P* < 0.001)

For determining whether the reduction of NO induced by HN was dependent on NOS or not, immunohistochemistry for iNOS was done. Sham group showed little positive cytoplasmic immunoreactivity for iNOS in the villi core and the lining enterocytes (Fig. 4a), while the I/R group showed extensive positive iNOS immunoreactivity (Fig. 4b). Moreover, the HN-I/R group showed moderate cytoplasmic immunoreactivity for iNOS (Fig. 4c).

Morphometric analysis for area percentage of iNOS immunoreactivity in the I/R group, there was a significant increase vs sham group (*p* < 0.001). While the HN-I/R group showed a significant increase of iNOS immunoreactivity vs the sham group (*p* < 0.01) and a significant decrease vs the I/R group (*p* < 0.001) (Fig. 4d).

### Effect of humanin on small intestinal motility

**Fig. 5:**
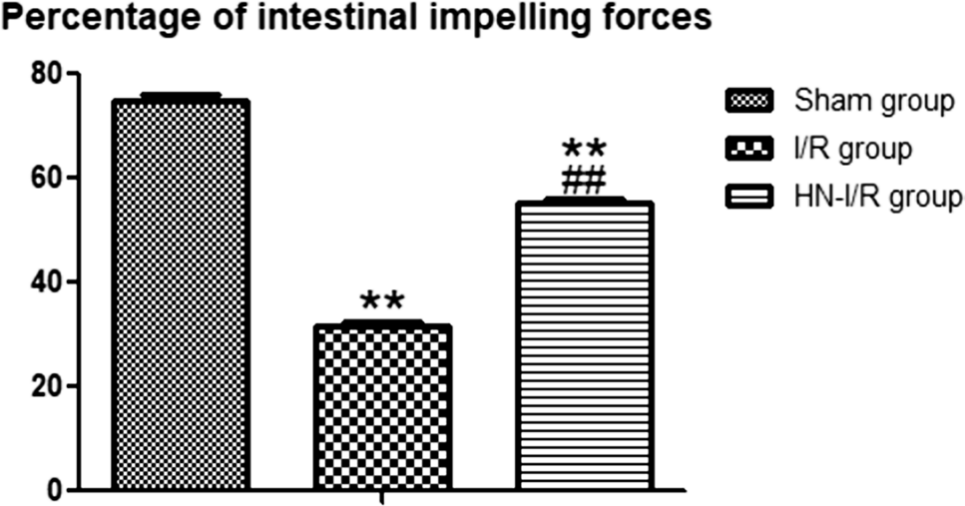
Percentage of intestinal impelling forces in all studied groups: Data are presented as mean ± SD, ^**^: highly significant versus sham group (*P* < 0.01), and.^##^: highly significant versus I/R group (*P* < 0.001)

It was found that the percentage of intestinal impelling force was significantly declined in the I/R group (*P* < 0.001), as compared to the sham group, as the distance traveled by the charcoal meal in the I/R group was markedly decreased. However, the percentage of intestinal impelling force was significantly increased in the HN-I/R group, as compared to the I/R group (*P* < 0.001) (Fig. 5).

## Discussion

An abrupt interruption of blood flow to the intestine, followed by blood flow reconstruction, causes oxidative stress, bacteria to go to the area of inflammation, and cells to respond by going through planned processes such as apoptosis, autophagy, and necroptosis [47]. One peptide produced by mitochondria called humanin has been linked to autoimmune disease, metabolic problems, and cardiovascular and other diseases in addition to ageing, through the control of several biological processes, such as autophagy, endoplasmic reticulum stress, cellular metabolism, oxidative stress, and inflammation [28].

Our experimental findings proved the protecting effect of humanin on a model of intestinal I/R injury in rats. We found that humanin treatment resulted in significant improvements of intestinal oxidant and inflammatory states. Interestingly, MDA as an important marker of lipid peroxidation, Il-6 and TNF-α as markers of inflammation were markedly increase with I/R injury and decreased with humanin treatment however the reverse occurred with antioxidant enzymes SOD and GPx levels.

MDA is one of the final products of polyunsaturated fatty acids peroxidation in the cells. An increase in free radicals causes the overproduction of MDA [65]. Many reports proved the oxidative stress and inflammatory process in intestinal I/R [47, 57, 68]. In addition, IL-6 and TNF-α activate NF-κB which is translocated to the nucleus for regulation of its target genes transcription, including inflammatory mediators, acute phase proteins, cytokines, and inducible effector enzymes [49].

It should be noted that numerous other researches have demonstrated the antioxidant properties of humanin in cardiomyocytes, cortical neurons, retinal pigment epithelium, and rat pancreatic beta cells [20, 53, 55]. Additionally, the humanin analogue reduced hydrogen peroxide production by 55% when given to isolated mitochondria from rat insulinoma (INS-1) beta cells, demonstrating that it has a cyto-protective function [53].

In this study, no histopathological changes were observed in the sham group. While the I/R group showed considerable damage of the jejunal mucosa. Degeneration and desquamation of jejunal villi, vacuolations, hemorrhage, and mononuclear cellular infiltration were observed. Similar histopathological findings were detected by Akdogan et al. [1] who induced ischemia–reperfusion injury by the Pringle maneuver and also by [34] who subjected rats to one or two hours of ischemia and subsequent reperfusion for 30 min. Additionally, jejunal villi were mainly affected especially their tip but jejunal crypts were apparently intact; this was parallel with the results of Scheer et al. [58] who induced mesenteric ischemia for 90 min followed by 120 min of reperfusion. Buyukhatipoglu et al. [9] caused the intestinal injury following reperfusion by increased ROS.

In the current study, the HN-I/R group showed pronounced improvement of histological signs, when compared to the I/R group. As, HN attenuated the severity of inflammation and cellular infiltration caused by ischemia and reperfusion. These results were parallel to the results of Gultekin et al. [32] who proved that HN attenuated colonic inflammation and ulceration stimulated by 2,4,6-trinitrobenzene sulphonic acid.

In this study, the I/R group showed a high significant decrease of jejunal villus length and a high significant increase in villus width, when compared with the sham group. These results were consistent with Guillaumon and Couto [31]. In contrast, the HN-I/R group exhibited improvement of jejunal villus width and length, when compared with the I/R group. This was in agreement with Zhu et al. [75], as HN defends cells from a variety of pathogenic situations, such as skeletal and neurological disorders.

Caspase-3 is thought to be the primary protein with apoptotic activity and functions as a junction between the endogenous and exogenous apoptotic pathways [48]. As a result, its expression levels can accurately predict when apoptosis occurs [43].

In the current work, the area percentage of caspase-3 immunoreactivity in the I/R group was significantly high, as compared to the sham group. This was in agreement with Akdogan et al. [1] and also with Takeshita et al. [63] who subjected rats to 30 min ischemia and subsequent reperfusion for 30 min.

In our study, HN-I/R groups showed a high significant decrease of the area percentage of caspase-3 immunoreactivity, when compared with the I/R group. Interestingly in our work, serum TNF-α significantly increased, together with increasing tissue expression of caspase-3 enzyme activity, which are important indicators of cell apoptosis. In the same line, ÇEVİK et al. [11] showed that TNF-α considerably stimulated cytochrome c cytosolic release, which participates in gene and protein expression of caspase-3.

These results were supported by a study on ulcerative colitis, Gultekin et al. [32] hypothesized that HN protected colonic epithelial cells by inhibiting apoptosis and inflammation; this was proved by diminishing mRNA expressions of caspase-3, TNF-α, and IL-1β activities in the colonic tissues. Moreover, Gottardo et al. [29] demonstrated that HN inhibited apoptosis induced by TNF- α in anterior pituitary cells of male and female rats that had undergone gonadectomies. A further support to our data, El Kattawy et al. [24] proved that HN has anti-aging properties and anti-apoptotic as HN elevated PCNA and BCL2 activity and reduced testicular caspase-3 activity and suppressed pro-apoptotic BAX protein expressions in the aged rats.

In the same line, the present study observed a significant elevation of intestinal NO levels in injured intestinal tissue of the I/R group. However, humanin treatment HN partially corrected the elevated NO. In a model of silver nanoparticles-induced neurotoxicity in human neuroblastoma cancer cells, Gurunathan et al. [33] verified that humanin decreases excessive NO levels in cultured neuron cells and protects brain cells [20, 50].

NO is produced by a class of enzymes called nitic oxide synthase (NOS). NOS has three main isoforms: neuronal nNOS, endothelial eNOS, and macrophage iNOS [69]. Endogenous NOS are represented as eNOS and nNOS [38]. Endogenous NO shields the intestines against inflammation and injury brought on by hypoxia, according to research by Caplan et al. [10] on the subject. However, numerous papers claim intestinal inflammation brought on by the IR must elevate iNOS [18, 38, 69].

Therefore, it was necessary for us to search for more relations between HN and NO and search for mechanisms behind increased NO levels in this model, so we did the immunohistochemical study for iNOS activity in intestinal tissue. We found that the area percentage of iNOS immunoreactivity in the I/R group was considerably high, as compared to the sham group. On the other hand, the HN-I/R group displayed a high significant decrease of the area percentage of iNOS immunoreactivity, when compared with I/R group.

Notably, iNOS does not express itself much at baseline until an immunological response, cytokines and oxidative stress activate iNOS [44, 70]. Because iNOS is responsible for the creation of massive bursts of NO during I/R, several studies have hypothesized that NO produced from iNOS is hazardous in I/R [14, 66]. Additionally, Chatterjee et al. [13] have indicated that selective iNOS inhibition and subsequent decrease in NO generation can minimize renal injury and dysfunction related to renal I/R.

According to a study on cardiac I/R, iNOS/NO-mediated factors, such as the formation of oxy-radicals like peroxynitrites, have cytotoxic effects on nearby cardiomyocytes due to the start of lipid peroxidation, inhibition of mitochondrial respiratory chain enzymes, and release of iron from ferritin, and also contribute to cardiac dysfunction after infarction [36, 70]. Additionally, in human inflammatory bowel disease, up-regulation of NO production through iNOS expression has also been linked to the start and maintenance of inflammation [41].

From another point of view, numerous evidence lines point to the possibility of NO to control the expression of certain genes linked to apoptotic stimuli [5, 26, 27, 52]. Also, Yang et al. [44] found that, through multiple signal transduction pathways, iNOS overexpression causes cell death and exacerbates lung damage. Furthermore, Kiang et al. [40] established that iNOS expression levels and caspase-3 protein levels are linked, detected by western blot analysis in intestinal epithelial cells with iNOS gene transfection, and it was found that, in the iNOS gene of transfected cells, the iNOS overexpression is accompanied by an increase in caspase-3 activity. Additionally, in human platelets, iNOS increases NO production and controls caspase-3 activation [11].

In another view, the present study examined how I/R injury affects the intestinal motility, through measuring the intestinal transit time using a charcoal meal. Interestingly, there was a significant decrease in intestinal impelling force in the I/R group when compared with the sham group. However, the intestinal impelling force was significantly improved in the HN-I/R group, when compared with the I/R group. I/R motility alterations may be produced by the hypoxia and oxidative stress and subsequent apoptosis occurring in our model. Bielefeldt and Conklin [7] also referred to the motility changes in intestinal ischemia to a depletion of energy and the hypoxic damage to smooth muscle cells. And this may explain the improvement in the HN-I/R group.

In addition, the elevated inflammatory mediators in our model are partially implicated in this motility disturbance, as intestinal I/R cause the ischemic gut to become inflamed [17, 74]. According to reports, inflammatory mediators and their metabolites may alter neuromotor function, which would alter motility [3, 22, 42, 59]. These inflammatory processes may also impact other organs in addition to the intestine [45].

Notably, some gastrointestinal motor dysfunctions have been attributed to alter NO generation or release [21, 30]. Selective iNOS inhibition has been proposed as a therapy method for this potentially fatal illness, and it has been postulated that a toxic megacolon in individuals with ulcerative colitis may be generated by an excess of NO produced by iNOS in the colonic smooth muscles [45]. Colonic hypomotility seen in induced chronic pancreatitis has also been reported to be caused by elevated iNOS in the myenteric plexus, thus, proving the contribution of NOS in the pathogenesis of colonic dysmotility [15]. In addition, Takahashi et al. and also Taha et al. [61, 62] demonstrated that injection of N(G)-Nitro-L-arginine methyl ester (L-NAME) which is NOS inhibitor caused a progressive rise in jejunal motility and an increase in contraction amplitude.

## Conclusion

We confirmed a new beneficial effect for humanin on intestinal I/R injury through its antioxidant, anti-inflammatory, and antiapoptotic effects in addition to its suppression effect on iNOS/NO pathway.

Based on our study and other studies, there is a possible relation between iNOS/NO pathway and caspase-3. However, the relation between iNOS/NO pathway and apoptotic markers is still unclear and needs more future studies to be declared. We can conclude that the biological NO regulates motility, blood flow, and oxygen uptake and that these NO-related regulations are affected by one another and the activity of NO depends on the nitric oxide source, local concentration, and the presence of other reactive molecules that can control the function of NO. And this suppressor effect of humanin on iNOS may be considered as a novel indirect antiapoptotic mechanism of humanin.

We suggested that humanin did not suppress constitutional eNOS and nNOS which are beneficial and essential for basic physiologic function, as HN partially corrected the elevated NO, it did not decrease its levels below normal; however, we recommend further study to determine the possible relation between HN and these two NOS isoforms.

Additionally, for the first time, we confirmed a beneficial effect for humanin on I/R-associated small intestinal dysmotility by increasing intestinal impelling force. In line with the above-mentioned data, this beneficial effect on intestinal motility may be partly due to its antioxidant, anti-inflammatory, and antiapoptotic effects which have been proved in our model. However, interestingly, we should highlight on the suppressor effect of humanin on the expression of iNOS/NO which may be considered a novel mechanism for humanin in the reversal of I/R injury-induced small intestinal dysmotility, in addition to the possible use of humanin as a potential pharmacological agent in clinical application.

Finally, we recommend additional future in vitro studies on intestinal contractility using humanin and selective agonists and antagonists for NO action to approve the role of NO in intestinal dysmotility in the intestinal I/R model.

## Data Availability

The corresponding authors are willing to provide the data and datasets used and/or analyzed during the current study upon reasonable request.
